# An investigative study on *Yersinia enterocolitica* in animals, humans and dried milk in New Valley Governorate, Egypt

**DOI:** 10.1186/s12866-024-03527-7

**Published:** 2024-10-09

**Authors:** Sotohy A. Sotohy, Mohamed Said Diab, Rania M. Ewida, Allaa Aballah, Nehal K. Alm Eldin

**Affiliations:** 1 Department of Animal, Poultry and Environmental Hygiene, Faculty of Veterinary Medicine, Assiut University, Asyut, Egypt; 2 Department of Animal Hygiene and Zoonoses, Faculty of Veterinary Medicine, New Valley University, Kharga Oasis, Egypt; 3 Department of Food Hygiene (Milk Hygiene), Faculty of Veterinary Medicine, New Valley University, El-Kharga, Egypt

**Keywords:** Antibiotic resistance, *Ail*, *16S rRNA*, *Yst* gene, Genetic similarity, Cattle *Yersinia enterocolitica*, Zoonoses

## Abstract

**Background:**

Yersiniosis is one of the most significant intestinal disorders caused by *Yersinia enterocolitica* and affects both humans and animals. This study aimed to investigate the prevalence of *Y. enterocolitica* in New Valley Governorate, Egypt in animals, humans, fresh milk and dried milk. Additionally, this study analyzed the presence of virulence genes, including *ail* and *Yst in* tested isolates and conducted a phylogenetic analysis to determine the genetic similarity between human, and animal *Y. enterocolitica* isolates. Finally, the antimicrobial resistance patterns of the isolates were examined.

**Results:**

Among the 982 samples examined, the prevalence of *Y. enterocolitica* based on ISO10273-2017 was 11.7% in animal samples including 12.8% of animal faeces, and 10.4% in milk samples. Moreover, the prevalence of *Y. enterocolitica* was 13.2% in human stool, and 9.5% in dried milk samples. The molecular characterization of the six randomly selected isolates showed that the *16S rRNA, ail* and *Yst* genes were found in 50, 33.3 and 100% of the examined *Y. enterocolitica* isolates*,* respectively. Phylogenetic analysis of animal and human isolates based on the *16S rRNA* gene revealed a high degree of similarity between the isolates. All the tested animal and human *Y. enterocolitica* isolates (100%) were resistant to ampicillin and cefotaxime, but highly sensitive to norfloxacin.

**Conclusions:**

The high prevalence of *Y. enterocolitica* in animal and human samples with high degrees of genetic similarity poses a threat to public and animal health. Animal faeces, milk and milk powder represent the main sources of *Y. enterocolitica* infection in humans. Additionally, high levels of antibiotic resistance of *Y. enterocolitica* can cause public health hazards by leading to the failure of disease prevention and treatment programs in humans and animals.

**Supplementary Information:**

The online version contains supplementary material available at 10.1186/s12866-024-03527-7.

## Background

Yersiniosis is one of the most significant intestinal disorders caused by *Y. enterocolitica* and affects humans and numerous animals including cattle, sheep, and goats [1, 2]. Many social and environmental factors contribute to the spread of *Y. enterocolitica* causing significant public health and economic problems [3, 4].

Despite the primary clinical signs of domestic animals being self-limiting (sialorrhea, diarrhea, and weight loss), recovered and asymptomatic animals can contaminate pastures and the surrounding environment via contaminated feces with *Y. enterocolitica* and the infection can spread through animals [1].

Human yersiniosis is food-borne as it is transmitted through contaminated meat, milk, and related products. Immunocompetent patients often present with self-limiting yersiniosis that manifests as enteritis and colitis with secretory diarrhea, fever, stomach pain, and emesis in rare cases [5], but *Y. enterocolitica* infection typically affects children, elderly people, and people with impaired immune systems and the clinical signs appear to be fever, vomiting, stomach pain, and bloody diarrhea [2].

Yersiniosis have significant economic impacts due to healthcare and veterinary costs, productivity and livestock losses, trade restrictions, food recalls, and the need for regulatory compliance and public health measures. In 2022, yersiniosis was the fourth most reported gastrointestinal infection in the EU/EEA, with 8,037 confirmed cases in 27 countries [6–8].

In addition to the traditional bacteriological examination, a variety of genotypic techniques have been developed for the confirmation of *Y. enterocolitica*. The *Y. enterocolitica 16S rRNA*-based PCR technique is mostly used for confirmation of this bacterium. *Y. enterocolitica* pathogenicity depends on the expression of chromosome and plasmid determinants. *Y. enterocolitica* virulence genes provide genetic information and plays a key role in reaching the upper part of the large intestine, rapid proliferation, colonization, and infection. Only the pathogenic strains of *Y. enterocolitica* have virulence genes, such as the chromosomal virulence genes *ail,* and *Yst,* in their genome [2, 9]. The *ail* gene encodes a protein that plays an essential role in the adhesion and invasion of host cells. Additionally, the *yst* gene encodes a heat-stable enterotoxin that is responsible for host diarrhea [7, 10].

Antimicrobial resistance is a serious international problem that restricts the management of numerous bacterial infections in the veterinary and general health care fields [11]. The use of antibiotics is a common practice in the treatment of bacterial diseases in animals and humans. Unfortunately, the use of antibiotics is not controlled by any laws in Egypt, so antimicrobials are widely used as growth promoters and feed additives for animals, which leads to the persistence of antibiotic residues in meat and eggs and increases the development of multidrug-resistant bacteria. Moreover, the continuous use of the same types of antibiotics and the misuse of antibiotics and subtherapeutic doses contribute to the development of resistant bacteria that can transfer this resistance to other bacterial strains [12, 13]. Antimicrobial resistance is the main cause of treatment failure and increases the morbidity and mortality of humans and animals. It is crucial to decrease the use of antibiotics as much as possible and use new antimicrobial drugs to decrease the risk of antibiotic-resistant-bacteria to preserve public health [14, 15].

The primary goal of this research was to collect information regarding the occurrence of *Y. enterocolitica* in both animals and humans, as well as in dried milk products, within the New Valley governorate, Egypt. Additionally, this study aimed to assess the pathogenicity of the isolates by detecting the *ail* and virulence genes. Furthermore, the genetic similarity between human and animal *Y. enterocolitica* isolates was determined through phylogenetic analysis. Finally, the antimicrobial resistance patterns of *Y. enterocolitica* isolates to the most commonly used antibiotics in the study region were analyzed.

## Results

A total of 982 samples from raw milk, animal feces, dried milk and human stool were examined for the presence of *Y. enterocolitica*. The results in Table 1 show that the prevalence of *Y. enterocolitica* was 11.7 (60/512), 13.2 (18/136) and 9.6% (32/334) in the animal, human and dried milk samples respectively. The prevalence of *Y. enterocolitica* was 12.8 (36/282) and 10.4% (24/230) in animal feces, and fresh milk samples, respectively, as shown in Table 1. The results showed that although the isolation rate is higher in the stool than fresh milk, there are no significant differences. While the statistical analysis showed Significant differences in the isolation rate between powdered milk and infant formula.

**Table 1:** Overall prevalence of *Y. enterocolitica* in animal, human and dried milk samples

Sample		No	Positive No	Positive %	Chi-square	*P*-value
Animal samples	Fresh Milk	230	24	10.4	0.391	0.532
	Faeces	282	36	12.8		
Subtotal		512	60	11.7		
Human samples	Stool	136	18	13.2		
Dried milk	Milk powder	40	8	20	5.14	0.023
	Infants’ formula	294	24	8.2		
Subtotal		334	32	14.1		
Total		982	110	11.2		

Non-significant (*P* > 0.05)

Six randomly selected isolates were tested with PCR for the presence of the *16S rRNA* gene “two isolates of animal feces in addition to one each of fresh milk, powdered milk, infant formula and human stool”. The results revealed that the *16S rRNA* gene was found in 50% of the isolates*.* The positive *16S rRNA* isolates were tested for the presence of the *ail* and *Yst* genes, and 33.3 and 100% of the examined *Y. enterocolitica* isolates had the *ail* and *Yst* genes*,* respectively, as shown in Table 2.

**Table 2:** Prevalence of *16S rRNA, ail* and *Yst* genes of some *Y. enterocolitica* isolates

Gene	Sample No	+ ve No	+ ve %
*16S rRNA*	6	3	50
*ail*	3	1	33.33
*Yst*	3	3	100

**Fig. 1:**
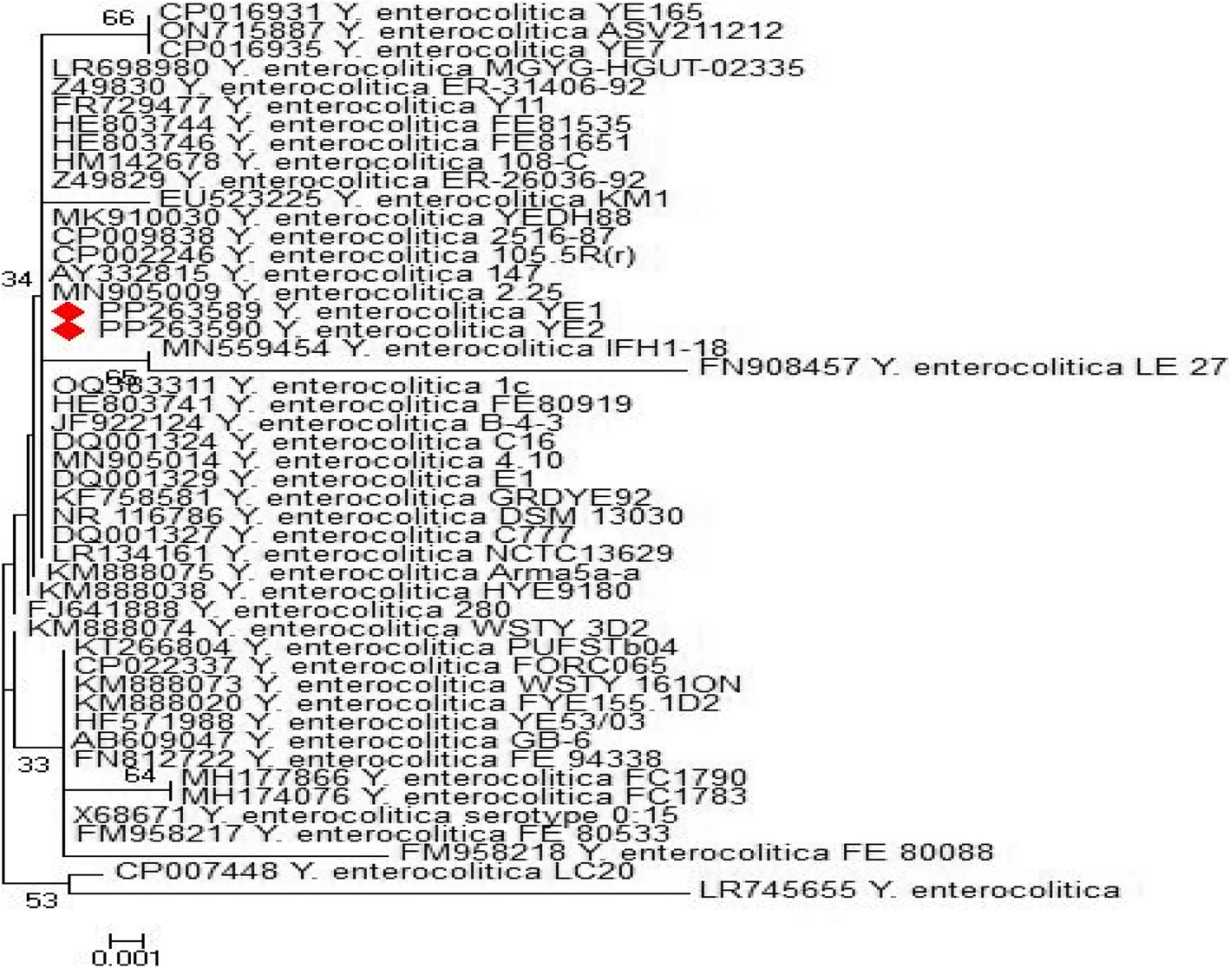
Phylogenetic tree of human and animal *Y. enterocolitica* isolates based on *16 s rRNA* gene

**Fig. 2:**
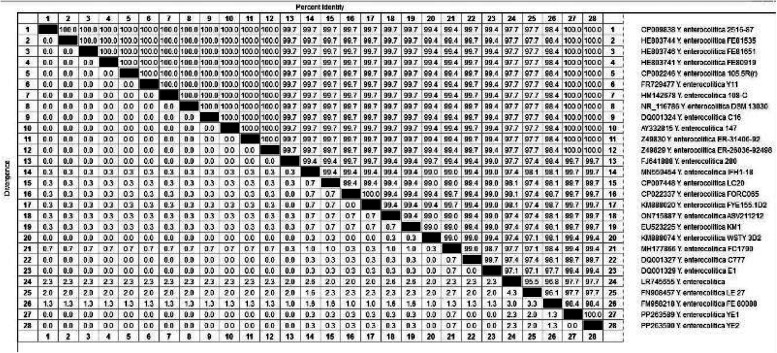
File distance of human and animal *Y. enterocolitica* isolates based on *16srRNA* gene sequencing

As shown in Figs. 1 and 2, sequencing of the *16S rRNA* gene from the animal and human isolates revealed 100% identity between the isolates and also with many isolates in GenBank, the accession number of the deposited animal isolates in GenBank is PP263589, and that for the human stool isolates is PP263590.

Overall, 110 *Y.enterocolitica* isolates were tested for antibiotic susceptibility to the most commonly used antibiotics in the study area. The data in Table 3 illustratethat the animal *Y. enterocolitica* isolates showed the highest resistance (100%) to ampicillin and cefotaxime followed by streptomycin (83.3%). In contrast, the animal *Y. enterocolitica* isolates showed the highest sensitivity to norfloxacin.

**Table 3:** Antibiotic sensitivity of isolated *Y. enterocolitica* from animal's samples

Antimicrobial agent	S		I		R	
	**NO**	**%**	**NO**	**%**	**NO**	**%**
**Ampicillin (AMP10)**	0	0	0	0	60	100
**Gentamicin (GN10)**	40	66.66	0	0	20	33.33
**Azithromycin (AT15)**	30	50	0	0	30	50
**Streptomycin (S10)**	10	16.66	0	0	50	83.33
**Chloramphenicol (C30)**	40	66.66	0	0	20	33.33
**Norfloxacin (NOR10)**	60	100	0	0	0	0
**Cefotaxime (Cxm30)**	0	0	0	0	60	100

As shown in Table 4, the human *Y.enterocolitica* isolates presented the highest resistance (100%) to ampicillin and cefotaxime, followed by streptomycin, and chloramphenicol (61.1%, each), wheras the human *Y. enterocolitica* isolates exhibited moderate sensitivity to gentamicin and norfloxacin (61.1%, each).

**Table 4:** Antibiotic sensitivity of isolated *Y. enterocolitica* from, human samples

**Antimicrobial agent**	**S**		I		**R**	
	**NO**	**%**	**NO**	**%**	**NO**	**%**
**Ampicillin (AMP10)**	0	0	0	0	18	100
**Gentamicin (GN10)**	11	61.11	0	0	7	38.88
**Azithromycin (AT15)**	7	38.88	0	0	11	61.11
**Streptomycin (S10)**	7	38.88	0	0	11	61.11
**Chloramphenicol (C30)**	7	38.88	0	0	11	61.11
**Norfloxacin (NOR10)**	11	61.11	0	0	7	38.88
**Cefotaxime (Cxm30)**	0	0	0	0	18	100

According to Table 5, the antibiotics to which *Y. enterocolitica* isolated from dried milk was most resistant were ampicillin and cefotaxime (100% each). In contrast, the *Y. enterocolitica* strains isolated from dried milk presented the highest sensitivity to chloramphenicol (100%), followed by azithromycin (75%) and norfloxacin (75%).

**Table 5:** Antibiotic sensitivity of isolated *Y. enterocolitica* from dried milk samples

Antimicrobial agent	S		I		R	
	**NO**	**%**	**NO**	**%**	**NO**	**%**
**Ampicillin (AMP10)**	0	0	0	0	32	100
**Gentamicin (GN10)**	16	50	0	0	16	50
**Azithromycin (AT15)**	24	75	0	0	8	25
**Streptomycin (S10)**	16	50	0	0	16	50
**Chloramphenicol (C30)**	32	100	0	0	0	0
**Norfloxacin (NOR10)**	24	75	0	0	8	25
**Cefotaxime (Cxm30)**	0	0	0	0	32	100

Table 6 shows that the MAR index was greater in human samples (0.688) than in animal (0.538) or dried milk samples (0.475). The MAR index in the tested isolates ranged from 0.29 to 1.

**Table 6:** MAR index of *Y. enterocolitica* isolates

Samples	MAR index	Mean
Animals	0.523	0.562
Dried milk samples	0.475	
Human samples	0.688	

## Discussion

*Y. enterocolitica* is a significant zoonotic foodborne pathogen as it causes many disorders in animals and humans [16].

This study revealed that the prevalence of *Y. enterocolitica* was 11.7% in animals, which was higher than that reported in a previous study in Egypt (9.2%) [17]. However, it was lower than the prevalence of yersiniosis recorded in France (14.5%) [18]. A variety of factors such as environmental conditions, stocking density, the possibility of feed/water contamination, and the development of host immunity, can all contribute to the spread of *Yersinia* spp. among farm animals [19]. On the other hand, the prevalence of *Y. enterocolitica* in human samples was 13.24%, which was lower than that previously reported in Egypt (50%) [20], although it was higher than the prevalence recorded in Argentina 0.6% [21]. Additionally, the prevalence of *Y. enterocolitica* in dried milk samples was 9.6%, so children who consume these products are more susceptible to infection, especially since they are immunocompromised [22]. Moreover, the present data revealed that the prevalence of *Y. enterocolitica* in humans was greater than that in animals and dried milk samples, but the difference was not statistically significant (*P value* = 0.819).

The prevalence of *Y. enterocolitica* in fresh milk samples was 10.4%. A similar prevalence of 12% was previously reported in Iraq [23]. Different prevalence rates ranging from 0.83 to 33% have been reported in studies in Iran [24, 25]. Generally, milk is a favorable substrate for bacterial growth and multiplication, and whole milk may increase the growth of *Y. enterocolitica* at 3 °C [26].

The prevalence of *Y. enterocolitica* in faecal samples was 12.8%, which was higher than the previously reported in Sharqia Governorate, Egypt (2.8%) [26]. Our results also confirmed that the prevalence of *Y. enterocolitica* in faecal samples was greater than that in milk samples, but there was no statistically significant difference between the two results (*P value* = 0.532). Faeces and milk are the main sources of infection for humans with *Y. enterocolitica,* as the pathogen is found in the digestive tracts of many different animal species and is extensively spread throughout the environment via faeces*.* Consequently, *Y. enterocolitica* is present in raw milk samples taken from a variety of animal species as a result of faecal contamination during milking [2, 27].

**Fig. 3:**
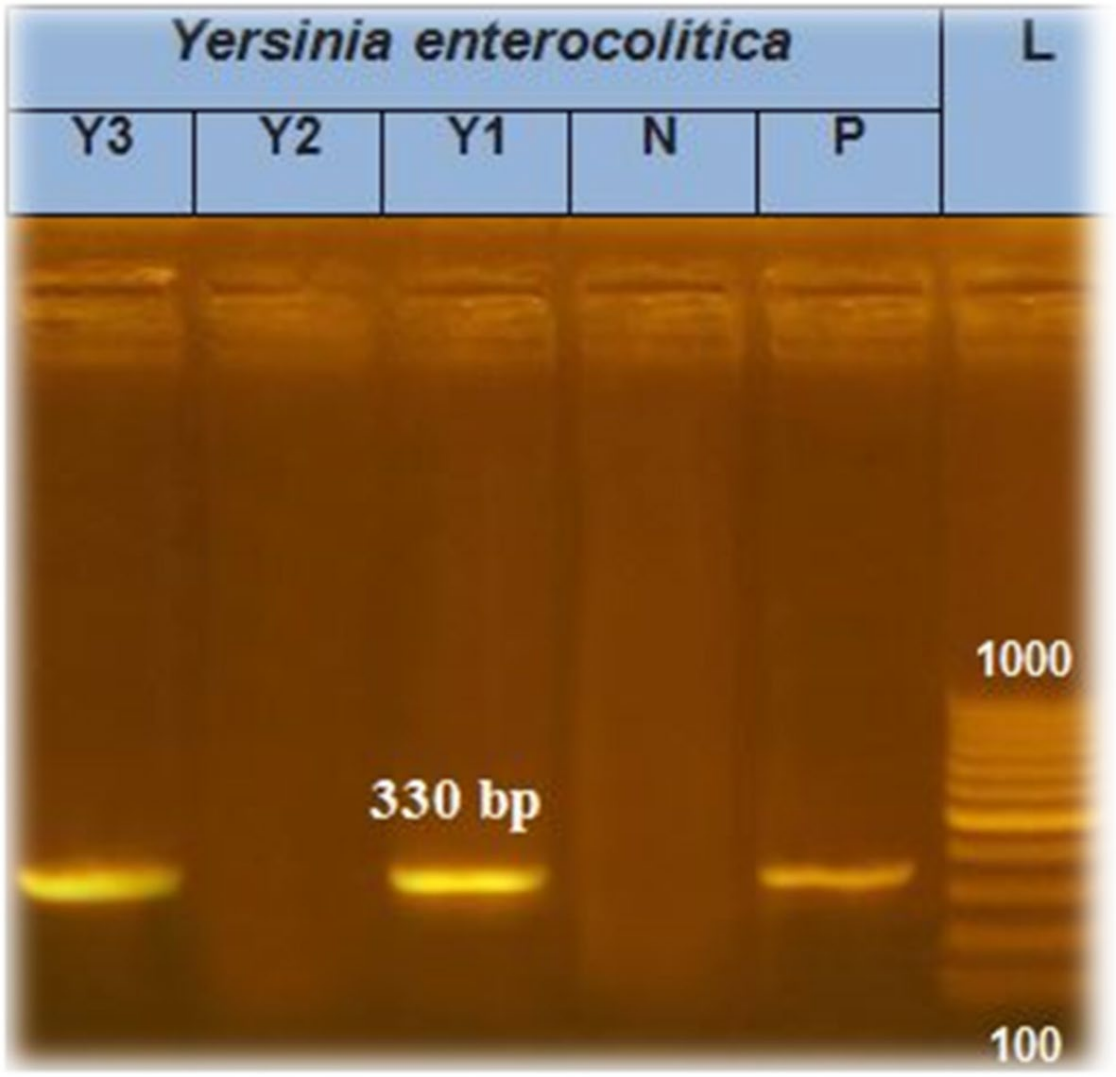
Agarose gel electrophoresis of amplified *16srRNA* of *Y. enterocolitica* gene

**Fig. 4:**
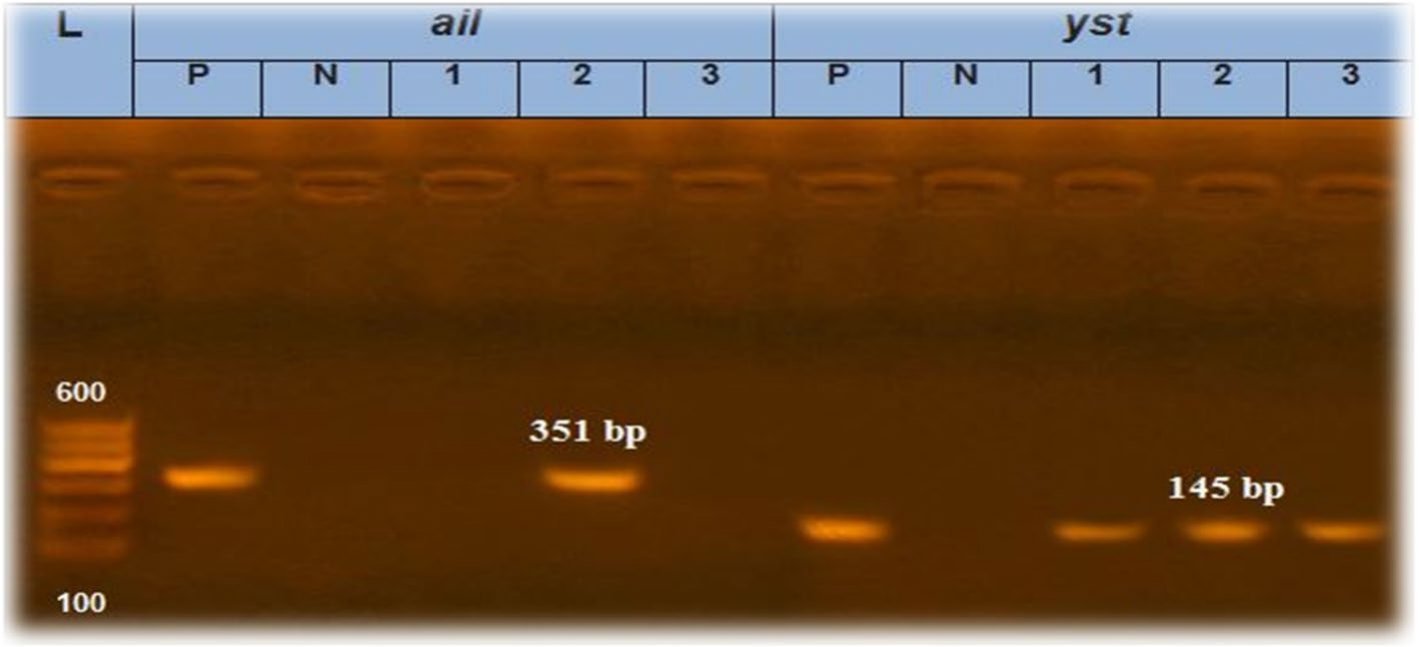
Agarose gel electrophoresis of amplified *ail and Yst* gene

Molecular identification experiments detected *16S rRNA* in 50% of the examined *Y. enterocolitica* isolates Fig. 3, in line with the findings of a previous study in Egypt [28]. On the other hand, the *16sr RNA* gene was detected in 26% and 12% of *Y. enterocolitica* isolates, respectively [23, 29]. Conversely, our results were lower than the results reported by Movafagh, et al. [24] who reported that *16srRNA* was detected in 94% of *Y. enterocolitica* isolates. Additionally, examination of *Y. enterocolitica* isolates revealed the presence of the *ail* and *Yst* genes in 33.3 and 100% of the samples Fig. 4*,* respectively. The findings of the present study aligned with those of a previous study that reported similar results for the *ail* gene (30%), although our results for the *Yst* gene were greater (10%) [26].

The data presented in Figs. 1 and 2 clarify that the sequencing of the *16S rRNA* gene in the animal and human isolates revealed 100% identity between the two examined isolates. Additionally, analysis revealed that, our isolates had a very high genetic similarity with isolates in GenBank from different sources such as pork products, fish intestine, meat and human stool (GenBank accession numbers KM888020- MN559454- EU523225- HE803744). This high level of genetic similarities between *Y. enterocolitica* of human and animal origin emphasizes the zoonotic role of this pathogen [8, 30].

Antimicrobial resistance is a serious international problem, so in the current study, antibiotic resistance *Y. enterocolitica* isolates from different sources were investigated. *Y. enterocolitica* of animal origin exhibited the highest resistance (100%) to ampicillin and cefotaxime followed by streptomycin (83.3%). These results were in agreement with those of previous study confirming the same results [31], but the current results differ with other studies that prove that *Y. enterocolitica* animal isolates were very sensitive to ampicillin and streptomycin cefotaxime [32, 33]. Our results revealed that the animal *Y. enterocolitica* isolates presented a relatively high resistance to chloramphenicol and gentamicin (66.66%, each). However, high sensitivity of animal *Y. enterocolitica* isolates to chloramphenicol and gentamicin has been reported in previous studies in Egypt [9, 34]. In the present study, the animal *Y. enterocolitica* isolates presented the highest sensitivity to norfloxacin, so it is the drug of choice for the treatment of diseased animals. This finding was compatible with a previous findings in Egypt [17, 35].

The human *Y.enterocolitica* isolates were 100% resistant to ampicillin and cefotaxime. The present results were consistent with those of several previous studies in Poland and Brazil [36, 37]. In contrast, our results were inconsistent with those of other studies showing that human *Y. enterocolitica* isolates are sensitive to cefotaxime [20, 38]. In the present study, the human *Y. enterocolitica* isolates presented moderate sensitivity to gentamicin and norfloxacin (61.11%, each), these results agreed with Nasser, et al. [20] who reported that the human *Y. enterocolitica* isolates showed a moderate sensitivity to gentamicin (57.9%).

*Y. enterocolitica* isolates of dried milk origin showed greater resistance to the antibiotics ampicillin and cefotaxime at 100%. This result was the same as that reported for the human and animal isolates in the present study. Additionally, these isolates showed intermediate resistance to gentamicin and streptomycin (50% each), However, these results differ from the results of animal and human isolates in this study. Animal isolates showed the highest resistance to streptomycin at 83.3%. While human isolates showed moderate sensitivity to gentamicin by 61.1%. Furthermore, the *Y. enterocolitica* isolated from dried milk had the highest sensitivity to chloramphenicol (100%), followed by azithromycin (75%) and norfloxacin (75%). These results contrast with the results of the animal and human strains in our study, as the animal and human *Y. enterocolitica* isolates presented relatively high resistance to chloramphenicol (66.7% and 61.1%, respectively).

The majority of the tested *Y. enterocolitica* isolates (80%) were MDR as they were resistant to more than three antibiotic classes. This high prevalence of MDR bacteria is an important concern for animals, and human health as it increases the cost of treatment, rate of hospitalization, and preventive strategies. Our result was higher than previous results in China and Egypt (23.3% and 27.5%, respectively [32, 39]. In contrast, our results were lower than those of some previous studies, which reported that 94.3%, and 92.3% of the tested *Y. enterocolitica* isolates were MDR, respectively [40, 41].

The MAR index was higher in human samples (0.688) than in animal (0.538) or dried milk samples (0.475). The MAR index ranged from 0.29 to 1. This high MAR index indicates an increase in drug resistance in the pathogen, which can be attributed to a variety of factors, such as improperly prescribed and illegal antibiotic use, prolonged use of antibiotics, and overuse of antibiotics as feed additives [42].

## Conclusion

The significant prevalence of *Y. enterocolitica* in both animal and human samples in the New Valley is alarming for both public and veterinary health. Animal faeces, fresh milk, and dried milk are an important sources of *Y. enterocolitica* infection to humans. Phylogenetic analysis of animal and human isolates revealed that they are 100% genetically identical emphasizing the zoonotic potential of *Y. enterocolitica*. Moreover, the high levels of antibiotic resistance of *Y. enterocolitica* can cause failure of prevention and treatment plans for this disease in both humans and animals leading to serious public health risks.

## Material and methods

### Ethical declaration

The study design was carried out according to the instructions of the Institutional Review Board of the Faculty of Medicine at Assiut University with Institutional Approval Number (04–2023-200252).

### Study area and design

**Fig. 5:**
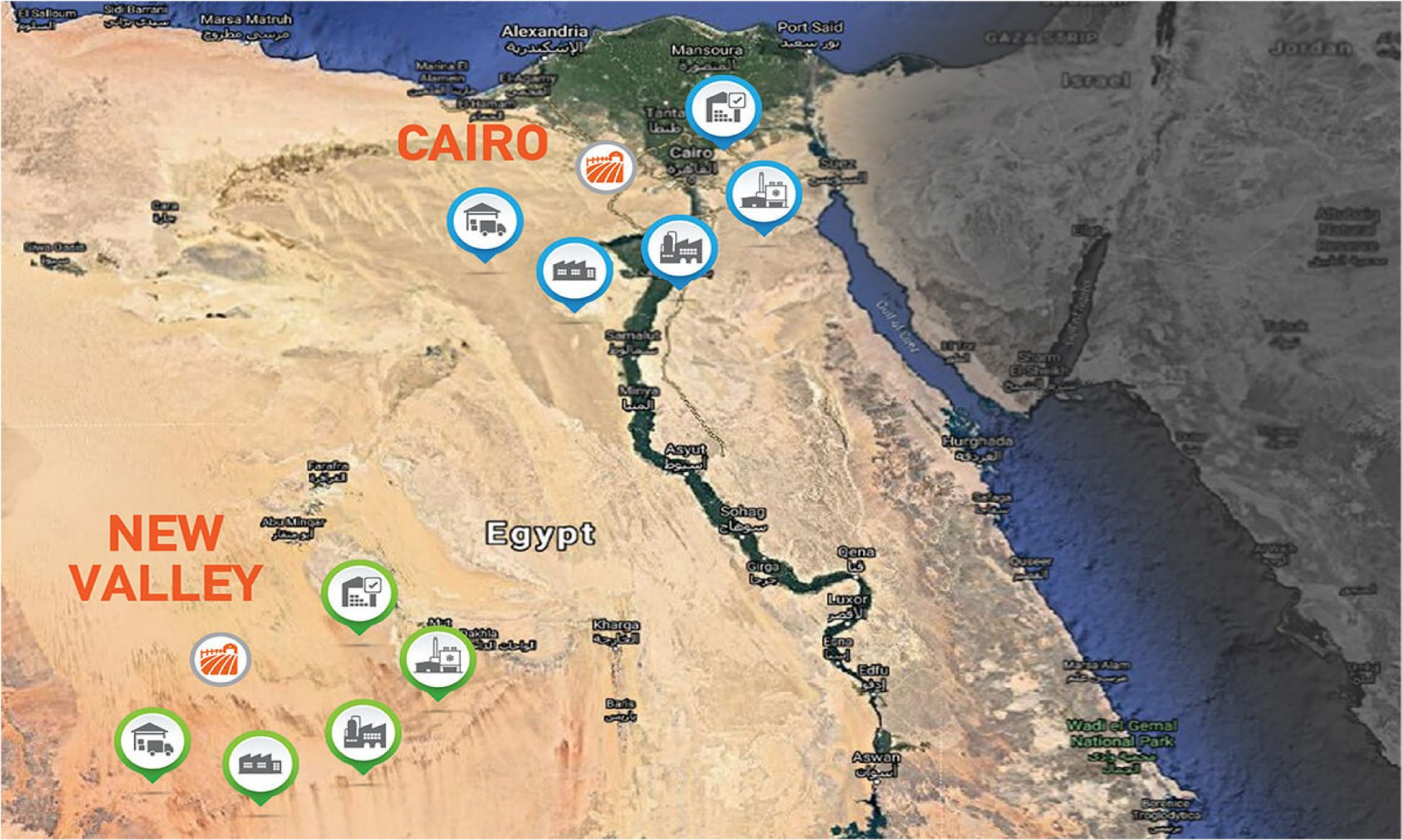
The locations of the study areas: New Valley Governorate, Egypt

The samples were collected from the largest Egyptian governorate, the New Valley governorate, from September 2022 to December 2023. New Valley is the largest Egyptian governorate located in the western desert of Egypt. The boundaries of the New Valley are shown in Fig. 5.

### Sampling

A total of 982 samples were collected including 230 fresh milk and 282 fecal samples from apparently healthy animals in the study area. In addition, 136 human stool samples were collected from different laboratories in EL Kharga. Moreover, 40 milk powder samples and 294 infant formula samples were collected from different supermarkets and houses.

### Bacteriological isolation and identification of *Y. enterocolitica*

The isolation of *Y. enterocolitica* was carried out according to the method of International Organization for Standardization ISO10273 [43] with slight modificaton. From each sample, one gram was added to nine mL of buffered peptone water (Himedia M614, India) and mixed well. One mL from the previous mixture was added to nine mL of enrichment broth in a tube containing phosphate-buffered saline supplemented with 0.15% bile salts and 1% sorbitol and incubated at 25 °C for three days. After enrichment, 0.5 mL of each tube was added to 4.5 mL of 0.5% KOH, then a loop-full of the mixture was streaked onto *Yersinia* selective agar base (Himedia M843, India) with *Yersinia* selective supplement (Himedia FD034, India) and incubated at 25 °C for 24 to 48 h. Suspected colonies with the characteristic red bull's eye appearance with a colorless translucent rim. Pure suspected colonies have been biochemically tested as they can ferment glucose, maltose, and mannitol, but they do not ferment lactose. Additionally, they are catalase positive, H_2_S and oxidase negative [9].

#### PCR technique for confirmation *Y. enterocolitica* isolates

PCR was applied to detect *16S rRNA* in randomly selected *Y. enterocolitica* isolates. In addition, the pathogenicity of the positive isolates was identified via the detection of the *ail* and *Yst* virulence gene using the primers shown in Table 7. *Y. enterocolitica* (ATCC 9610) was used as a positive control. The genomic DNA was extracted according to the QIAamp DNA mini kit instructions (catalogue no. 51304). The master mix used was the Emerald Amp GT PCR master mix (Takara, Code No. RR310A kit). The cycling protocol for the *16S rRNA* gene was carried out at 94 °C for 5 m for primary denaturation, followed by 35 cycles of 94 °C for 30 s for secondary denaturation, 62 °C for 40 s for annealing and 72 °C for 40 s for extension, followed by a final extension at 72 °C for 10 m. The PCR cycling protocol for the *ail* gene was 94 °C for 5 m for primary denaturation followed by 35 cycles of 94 °C for 30 s for secondary denaturation, 55 °C for 40 s for annealing, and 72 °C for 40 s for extension, followed by a final extension at 72 °C for 10 m. The PCR cycling protocol for the *Yst* gene was 94 °C for 5 m for primary denaturation, followed by 35 cycles of 94 °C for 30 s for secondary denaturation, 55 °C for 30 s for annealing, and 72 °C for 30 s for extension, followed by a final extension at 72 °C for 7 m.

**Table 7:** Oligonucleotide primers sequences

Agent	Target gene	Primers sequences	(bp)	Reference
***Y. enterocolitica***	***16S rRNA***	AAT ACC GCA TAA CGT CTT CG	330	[44]
		CTT CTT CTG CGA GTA ACG TC		
	***ail***	TAATGTGTACGCTGCGAG	351	[45]
		GACGTCTTACTTGCACTG		
	***Yst***	AATGCTGTCTTCATTTGGAGC	145	
		ATCCCAATCACTACTGACTTC		

#### Phylogenetic analysis

A comparative sequencing analysis of one animal and one human *Y. enterocolitica* isolate was performed using the *16S rRNA* gene. The *16S rRNA* gene sequence was determined with the CLUSTAL W multiple sequence alignment program and version 12.1 of the MegAlign module of Lasergene DNA Star Software Pairwise (Madison, Wisconsin, USA) according to Thompson, et al. [46], and phylogenetic analysis was performed via maximum parsimony in MEGA 6 [47].

### Antibiotic resistance of *Y. enterocolitica* isolates

The antibiotic sensitivity of the examined *Y. enterocolitica* isolates was measured on Mueller–Hinton agar (Himedia) via the disk diffusion method, following the instructions of the Clinical and Laboratory Standards Institute [48], and the interpretation of the inhibition zone diameters was carried out according to the clinical breakpoint value for Enterobacteriaceae. The diameters of the inhibition zones of the antimicrobial discs are shown in Table 8. The multi-antibiotic-resistance (MAR) index for each isolate was determined according to the following formula, where isolates classified as intermediate were considered sensitive based on the MAR index [49].

**Table 8:** Antimicrobial discs, concentration and interpretation of their action on the isolated Enterobacteriaceae

Antimicrobial agent	Disc content (*u*g)	Resistant (mm)	Intermediate (mm)	Sensitive (mm)
**Ampicillin (AMP10)**	10(ug)	≥ 13	14–16	≤ 17
**Gentamicin (GEN10)**	10 (ug)	≥ 13	13–14	≥ 15
**Azithromycin (AT15)**	15(ug)	≤ 12	_	≥ 13
**Streptomycin(S10)**	10(ug)	≤ 11	12–14	≥ 15
**Chloramphenicol(C30)**	30(ug)	≤ 12	13–17	≥ 18
**Norfloxacin (NX10)**	10(ug)	≤ 12	13–16	≥ 17
**Cefotaxime (Cxm30)**	30(ug)	≤ 22	23–25	≥ 26
Animal samples				
Human samples				
Dried milk				

( ≤) equal or less, ( ≥) equal or more

MAR index = No. of antibiotics with resistance / total no. of tested antibiotics.

### Statistical analysis

The statistical analysis was carried out using chi-square tests using SPSS, ver. 27 (IBM Corp. Released 2013). The data were treated as a complete randomization design according to Steel, et al. [50]. The significance level was set at < 0.05.

## Supplementary Information

Supplementary Material 1.

Supplementary Material 2.

## Data Availability

Sequences obtained were deposited in National Center for Biotechnology Information (NCBI) with accession numbers PP263589 and PP263590. (https://www.ncbi.nlm.nih.gov/nuccore/PP263589.1?report=GenBank) (https://www.ncbi.nlm.nih.gov/nuccore/PP263590.1?report=GenBank). (https://www.ncbi.nlm.nih.gov/nuccore/PP263590.1?report=GenBank).
