# Supplementary material for: An investigative study on Yersinia enterocolitica in animals, humans and dried milk in New Valley Governorate, Egypt
Source: BMC Microbiol. 2024 Oct 9;24:395. doi: 10.1186/s12866-024-03527-7 (PMC11462700; doi:10.1186/s12866-024-03527-7)
Supplement: Supplementary file 2 — Supplementary Material 2. [file 12866_2024_3527_MOESM2_ESM.pdf]

An official website of the United States government
Here's how you know

Log in

Nucleotide

GenBank

Yersinia enterocolitica strain YE2 16S ribosomal RNA gene, partial sequence

GenBank: PP263590.1

FASTA Graphics

Go to:

```
LOCUS       PP263590                 309 bp    DNA     linear   BCT 06-FEB-2024
DEFINITION  Yersinia enterocolitica strain YE2 16S ribosomal RNA gene, partial
            sequence.
ACCESSION   PP263590
VERSION     PP263590.1
KEYWORDS    .
SOURCE      Yersinia enterocolitica
  ORGANISM  Yersinia enterocolitica
            Bacteria; Pseudomonadota; Gammaproteobacteria; Enterobacterales;
            Yersiniaceae; Yersinia.
REFERENCE   1  (bases 1 to 309)
  AUTHORS   Diab,M.S., ALaa,M.A., Nehal,K.A. and Sotohy,A.A.
  TITLE     Molecular Detection of Yersinia enterocolitica from animal and
            human in New Valley Governorates
  JOURNAL   Unpublished
REFERENCE   2  (bases 1 to 309)
  AUTHORS   Diab,M.S., ALaa,M.A., Nehal,K.A. and Sotohy,A.A.
  TITLE     Direct Submission
  JOURNAL   Submitted (01-FEB-2024) Department of Animal Hygiene and Zoonoses,
            Faculty of Veterinary Medicine, New Valley University, km 10
            El-kharga, Assiut road, New-Valley 72511, Egypt
COMMENT     Sequences were screened for chimeras by the submitter using chimera
            check 3.

            ##Assembly-Data-START##
            Sequencing Technology :: Sanger dideoxy sequencing
            ##Assembly-Data-END##
FEATURES             Location/Qualifiers
     source          1..309
                     /organism="Yersinia enterocolitica"
                     /mol_type="genomic DNA"
                     /strain="YE2"
                     /isolation_source="New-valley"
                     /host="Homo sapiens"
                     /specimen_voucher="Stool"
                     /db_xref="taxon:630"
                     /collection_date="Dec-2023"
     rRNA            <1..>309
                     /product="16S ribosomal RNA"
ORIGIN
        1 taacgtcttc ggaccaaagt gggggacctt cgggcctcac gccatcggat gtgcccagat
       61 gggattagct agtaggtggg gtaatggctc acctaggcga cgatccctag ctggtctgag
      121 aggatgacca gccacactgg aactgagaca cggtccagac tcctacggga ggcagcagtg
      181 gggaatattg cacaatgggc gcaagcctga tgcagccatg ccgcgtgtgt gaagaaggcc
      241 ttcgggttgt aaagcacttt cagcgaggag gaaggcataa aggttaataa cctttgtgat
      301 tgacgttac
//
```
